# Identification of consistent QTL for time to maturation in Virginia-type Peanut (*Arachis hypogaea* L.)

**DOI:** 10.1186/s12870-021-02951-5

**Published:** 2021-04-19

**Authors:** Srinivas Kunta, Sara Agmon, Ilan Chedvat, Yael Levy, Ye Chu, Peggy Ozias-Akins, Ran Hovav

**Affiliations:** 1 Department of Field Crops, Agriculture Research Organization-The Volcani Center, Institute of Plant Sciences, HaMakkabbim Road, P. O. Box 15159, 7505101 Rishon LeZiyyon, Israel; 2 Faculty of Agricultural, Food and The Environmental Quality Sciences, The Hebrew University of Jerusalem, POB 12, 76100 Rehovot, Israel; 3 Department of Horticulture and Institute of Plant Breeding, Genetics and Genomics, University of Georgia, Tifton, GA 31793 USA

## Abstract

**Background:**

Time-to-maturation (TTM) is an important trait contributing to adaptability, yield and quality in peanut (*Arachis hypogaea* L). Virginia market-type peanut belongs to the late-maturing *A. hypogaea* subspecies with considerable variation in TTM within this market type. Consequently, planting and harvesting schedule of peanut cultivars, including Virginia market-type, need to be optimized to maximize yield and grade. Little is known regarding the genetic control of TTM in peanut due to the challenge of phenotyping and limited DNA polymorphism. Here, we investigated the genetic control of TTM within the Virginia market-type peanut using a SNP-based high-density genetic map. A recombinant inbred line (RIL) population, derived from a cross between two Virginia-type cultivars ‘Hanoch’ and ‘Harari’ with contrasting TTM (12–15 days on multi-years observations), was phenotyped in the field for 2 years following a randomized complete block design. TTM was estimated by maturity index (MI). Other agronomic traits like harvest index (HI), branching habit (BH) and shelling percentage (SP) were recorded as well.

**Results:**

MI was highly segregated in the population, with 13.3–70.9% and 28.4–80.2% in years 2018 and 2019. The constructed genetic map included 1833 SNP markers distributed on 24 linkage groups, covering a total map distance of 1773.5 cM corresponding to 20 chromosomes on the tetraploid peanut genome with 1.6 cM mean distance between the adjacent markers. Thirty QTL were identified for all measured traits. Among the four QTL regions for MI, two consistent QTL regions (*qMIA04a,b* and *qMIB03a*,*b*) were identified on chromosomes A04 (118680323–125,599,371; 6.9Mbp) and B03 (2839591–4,674,238; 1.8Mbp), with LOD values of 5.33–6.45 and 5–5.35 which explained phenotypic variation of 9.9–11.9% and 9.3–9.9%, respectively. QTL for HI were found to share the same loci as MI on chromosomes B03*,* B05, and B06, demonstrating the possible pleiotropic effect of HI on TTM. Significant but smaller effects on MI were detected for BH, pod yield and SP.

**Conclusions:**

This study identified consistent QTL regions conditioning TTM for Virginia market-type peanut. The information and materials generated here can be used to further develop molecular markers to select peanut idiotypes suitable for diverse growth environments.

**Supplementary Information:**

The online version contains supplementary material available at 10.1186/s12870-021-02951-5.

## Background

Peanut (*Arachis hypogaea* L.) is an important grain legume and oilseed source for human nutrition. It is grown in more than 100 countries and plays a significant role in global trade. In peanut, as in other legume crops, the growing period, or the time-to-maturation (TTM), is an essential characteristic for adaptation and yield. Although TTM in peanut is influenced by environmental conditions and agricultural practices [1, 2], it has a substantial genetic component reflected by the wide range of TTM among varieties. TTM was one of the crucial traits selected during the several thousand years of domestication and diversification. Cultivated peanut is classified into two subspecies, i.e., *A. hypogaea* ssp*. hypogaea* comprising Virginia and Runner market-types and *A. hypogaea* ssp*. fastigiata* including Spanish and Valencia market-types. The two subspecies diverge in maturity level, flowering pattern, shoot determination, and plant architecture [3]. The ssp*. fastigiata* is characterized by early fruit maturation, sequential flowering pattern, determinate shoot formation and erect growth habit, whereas ssp*. hypogaea* is late in fruit maturation and exhibits an alternative flowering pattern and indeterminate spreading or bunch habit [4]. Early-maturation (90 to 120 days post-planting) is necessary for drought avoidance in areas with a short rainy season. For this reason, Spanish market-type peanuts are predominately grown in West Africa and India, where the drought stress level is high [5]. On the other hand, high-yielding but late-maturing Runner- and Virginia- market-type peanuts are widely grown in the USA and the Middle East, where irrigation is available to most farming areas.

Variation in TTM has also been found within the ssp*. hypogaea.* Cultivars within subsp*. hypogaea* are classified as early-maturing (130–140 DPP), medium-maturing (140–150 DPP), and late-maturing (150–170 DPP) (https://issuu.com/onegrower/docs/peanut_grower_2019_variety_guide). Developing early maturing Runner- and Virginia-type cultivars with improved yield and excellent agronomic characteristics has been an important objective of peanut breeding programs. Early maturity is essential in areas that suffer from limited water supply or by end-of-season cooler temperatures and early frosts [6] that might retard maturation, cause incomplete seed filling, lower the yield and grade or quality (including oleic to linoleic acid ratios) [7, 8].

Despite its evident impact, very little is known regarding the genetic control of TTM in peanuts. The maturity level of peanut was reported as a quantitative trait with low heritability [9, 10] and influenced by many genes and environmental factors [11, 12]. A few attempts to define QTL with a small effect for early-maturation were reported using breeding materials from *fastigiata X hypogaea* crosses [13–15]. The utility of low-density genetic maps in these studies was one of the limiting factors for QTL discovery. Domesticated peanut is a self-pollinated allotetraploid (AABB, 2n = 4*x* = 40), originating from two diploid progenitors, *A. duranensis* (AA, 2n = 2*x* = 20) and *A. ipaensis* (BB, 2n = 2*x* = 20) [16, 17]. Cultivated peanut has a narrow genetic base caused by the bottleneck of a single hybridization event that gave rise to this species and the crossing barrier between cultivated peanut and wild diploids due to ploidy differences [18]. With the advancement of SNP array technology [19, 20], the limitation of genetic map density was alleviated by a drastic increase in genetic markers for map construction. Close to 1000 SNP markers were placed on peanut linkage maps recently [21, 22]. In addition to the low polymorphism, another challenge is to perform phenotyping on TTM due to the unique underground formation of fruit, the indeterminate nature of pod formation, and the application of the commonly used but laborious and somewhat subjective hull-scrape method to determine pod maturity [23].

Identifying genetic mechanisms controlling TTM in peanuts has a significant practical and scientific impact. Translating major QTL controlling the trait to user-friendly marker platforms will enable marker-assisted selection (MAS) to accelerate breeding for early- or late-maturity. In addition, underlying genetic mechanisms can be further investigated based on QTL mapping discoveries.

In the present study, a SNP-based linkage map was constructed for a RIL population derived from Virginia market-type parents. QTL mapping with two-year field phenotyping data led to the discovery of two consistent QTL for TTM, for the first time reported for Virginia-type peanut.

## Results

### Phenotyping of the parents and the RIL population

**Fig. 1:**
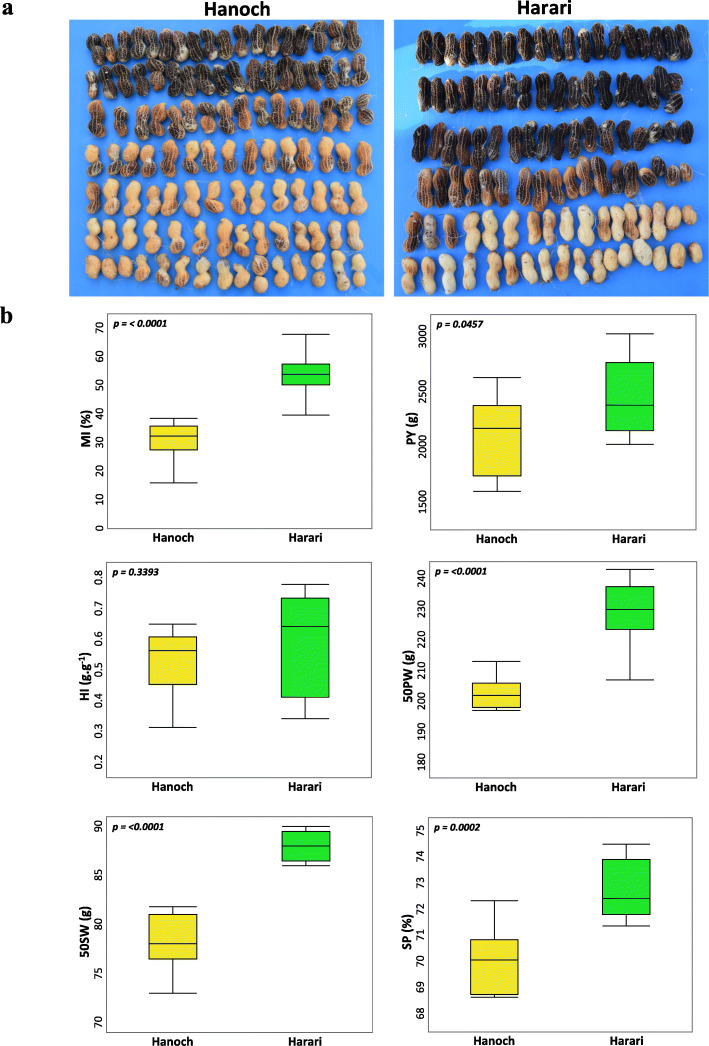
Phenotypic characterization of different traits among ‘Hanoch’ and ‘Harari’. **a**, MI morphology of ‘Hanoch’ and ‘Harari’. **b**, Comparisons between ‘Hanoch’ and ‘Harari′ in MI, PY, HI, 50PW, 50SW and SP. Data are shown as mean from two years (*n* = 9). The Student’s t-test was used to generate the P values. MI, maturity index (%); PY (g), pod yield; HI (g.g-1), harvest index; 50PW (g), 50 pod weight; 50SW (g), 50 seed weight; SP (%), shelling percentage

A RIL population was developed from a cross between Hanoch (late-maturing) and Harari (early-maturing) cultivars (Fig. 1a). The maturity index (MI), which is determined by the percentage of pods with black and brown mesocarp, was documented to indicate TTM. Data were collected from field experiments in two different environments (i.e., year, location, soil). MI values of parental lines were collected (Fig. 1b**)**. A highly significant difference was found between the parental lines in MI (*P* = < 0.0001), with 30.9 ± 6.82 and 53.97 ± 7.63 for Hanoch and Harari, respectively. In addition, significant differences were found between the parental lines for all of the other measured traits on the 2 years mean data, including pod yield (PY), 50-pod weight (50PW), 50-seed weight (50SW), and shelling percentage (SP), except for harvest index (HI) (Fig. 1b).

**Fig. 2:**
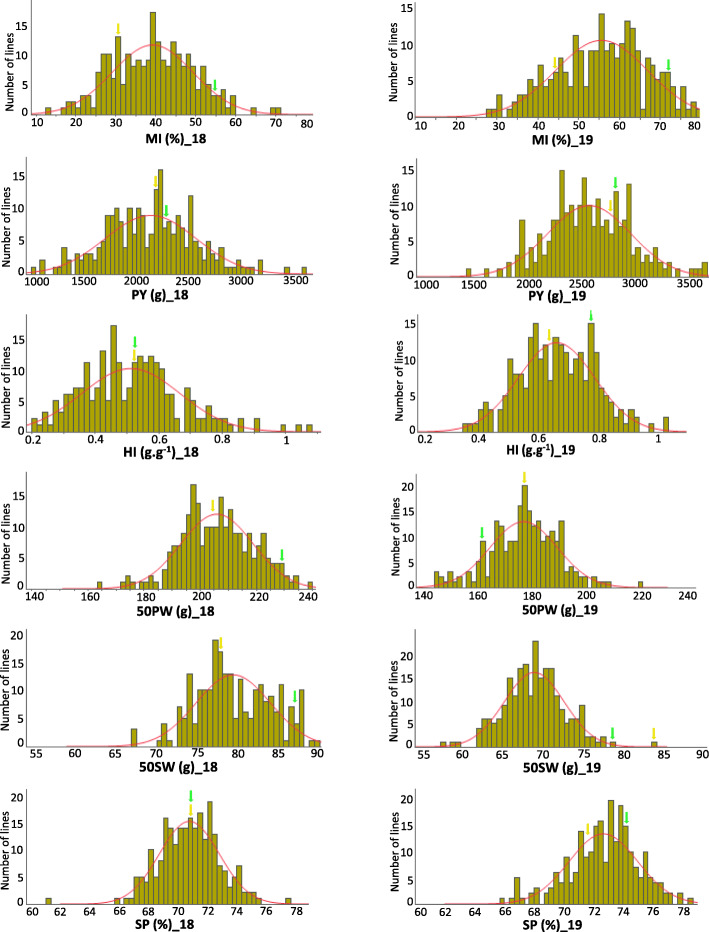
Phenotypic distribution of MI and the other traits in two consecutive years. Y-axis corresponds to the RIL population density, and X-axis corresponds to the original metric trait value based on the average of three replicates. Arrows indicate the phenotypic values for Hanoch (yellow) and Harari (green). A normal distribution curve is indicated in red. MI, maturity index; PY, pod yield; HI, harvest index; 50PW, 50 pod weight; 50SW, 50 seed weight; SP, shelling percentage. _18, year 2018; _19, year 2019

Normal or close to normal distribution was found in the RIL population data for all measured traits (Fig. 2; Table 1).

**Table 1:** Summary statistics of MI and other traits among parents and RILs

		Parents			RILs			
	variables	Hanoch	Harari	Student t- test	Mean ± SD	Minimum	Maximum	Sig. of A-D test^h^
2018	MI (%)^a^	30.9	53.9	< 0.0001	39.2 ± 10.1	13.3	70.9	0.583
	PY (g)^b^	2169.4	2274.9	0.7185^ns^	2142.1 ± 436.1	1037	3610	0.221
	HI (g.g^−1^)^c^	0.5	0.5	0.9794 ^ns^	0.5 ± 0.1	0.2	1.1	0.702
	50PW (g)^d^	203.4	229.2	< 0.0001	206.1 ± 12.8	163.5	238.8	0.336
	50SW (g)^e^	77.9	87.1	< 0.0001	79.4 ± 4.5	67	89.6	0.003^**^
	SP (%)^f^	70.7	70.9	0.7475 ^ns^	70.7 ± 2.1	61.1	77.6	0.638
2019	MI (%)	44.8	72.1	0.0022	55.6 ± 11.2	28.4	80.2	0.201
	PY (g)	2760	2813.3	0.8960 ^ns^	2566.5 ± 390.5	1453.3	3716.6	0.118
	HI (g.g^−1^)	0.6	0.8	0.0556 ^ns^	0.6 ± 0.1	0.3	1.1	0.002^**^
	50PW (g)	178.3	162.6	0.5177 ^ns^	177.3 ± 12.1	145	218.5	0.565
	50SW (g)	83.3	78.2	0.0098	68.8 ± 3.4	57.6	77.4	0.695
	SP (%)	71.6	74.1	0.9101 ^ns^	72.6 ± 2.3	65.7	78.4	0.014^*^

^a^ *MI* Maturity index; ^b^ *PY* Pod yield; ^c^ *HI* Harvest index; ^d^ *50PW* 50 pod weight; ^e^ *50SW* 50 seed weight; ^f^ *SP* Shelling percentage; ^h^ significance for normality test by Anderson-darling test; ^*^ and ^**^ mean significant at *P* < 0.05 and *P* < 0.01, respectively

Parental values of MI were within the range of the RILs. Some RILs exhibited a MI value beyond parental values at each end of the curve in both years, suggesting transgressive segregation of MI in this population. A significant effect was found for the blocks, RIL, year, and RIL X year interaction (Table 2) from ANOVA analysis. Therefore, QTL analysis was performed with data from each year separately. The broad-sense heritability for MI was 0.39, indicating a moderate-to-low but significant genetic component underlying this trait. The heritability estimates for other traits ranged from 0.07 (PY) to 0.36 (HI) (Table 2).

**Table 2:** Analysis of variance and heritability for MI and the other traits for the Hanoch X Harari RIL population across two years. Block [Year] indicates the nested effect of the Blocks within each year

Trait	Variables	DF	Mean square	F Ratio	*P*-Value	H^2 i^
MI ^a^	Block [Year]	4	3494.86	32.95	< 0.01	0.38
	Year	1	91,576.96	863.58	< 0.01	
	RIL	234	535.52	5.05	< 0.01	
	RIL x Year	234	139.71	1.31	< 0.01	
	Error	908	106.5			
PY ^b^	Block [Year]	4	4,208,786	12.83	< 0.01	0.07
	Year	1	55,806,460	170.22	< 0.01	
	RIL	234	483,510.34	1.47	< 0.01	
	RIL x Year	234	415,883.25	1.26	> 0.01	
	Error	785	327,833.75			
HI ^c^	Block [Year]	4	0.65	41.89	< 0.01	0.36
	Year	1	7.24	461.97	< 0.01	
	RIL	234	0.06	4.45	< 0.01	
	RIL x Year	234	0.02	1.53	< 0.01	
	Error	692	0.01			
50PW ^d^	Block [Year]	4	7777.31	52.39	< 0.01	0.33
	Year	1	276,752.62	1864.28	< 0.01	
	RIL	234	670.11	4.51	< 0.01	
	RIL x Year	234	236.63	1.59	< 0.01	
	Error	886	148.45			
50SW ^e^	Block [Year]	4	3057.62	191.09	< 0.01	0.3
	Year	1	37,556.15	2347.21	< 0.01	
	RIL	234	67.15	4.19	< 0.01	
	RIL x Year	234	27.65	1.72	< 0.01	
	Error	886	16.01			
SP ^f^	Block [Year]	4	1305.88	181.37	< 0.01	0.17
	Year	1	1263.04	175.43	< 0.01	
	RIL	234	17.52	2.43	< 0.01	
	RIL x Year	234	10.48	1.45	< 0.01	
	Error	887	7.19			

^a^ *MI* Maturity index; ^b^ *PY* Pod yield; ^c^ *HI* Harvest index; ^d^ *50PW* 50 pod weight; ^e^ *50SW* 50 seed weight; ^f^ *SP* Shelling percentage; ^i^ Broad sense heritability

**Fig. 3:**
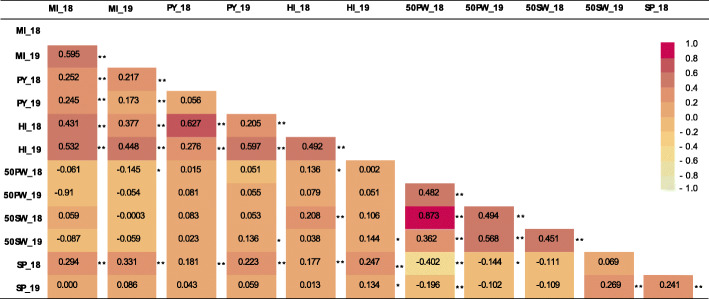
Pearson correlations for MI and the other traits evaluated in Hanoch X Harari RIL over two years. MI, maturity index; PY, pod yield; HI, harvest index; 50PW, 50 pod weight; 50SW, 50 seed weight; SP, shelling percentage. _18; year 2018; _19; year 2019. Significance of correlations: * *P* < 0.05 and ** *P* < 0.001

Pearson correlation among the traits was calculated in each year (Fig. 3). The correlation of the two-year MI measurements was 0.59 (*p* < 0.0001), suggesting a relatively higher genetic heritability of this trait than estimated by the ANOVA. MI was significantly correlated with HI and PY in both years and was correlated with SP in 2018. A small but significant correlation was found between MI in 2019 and 50PW in 2018. MI showed no correlation with 50SW. Significant correlations were observed among the other traits, such as SP with HI, SP with PY, and 50PW with 50SW. The Branching Habit (BH) phenotype effect (spreading vs. bunch) on MI was inspected by a T-test (Additional file 1: Fig. S1). A significant but small effect was found for the branching habit (BH) phenotype on MI in both years, in which spreading type lines had higher MI values than bunch types.

### Construction of the genetic map

**Fig. 4:**
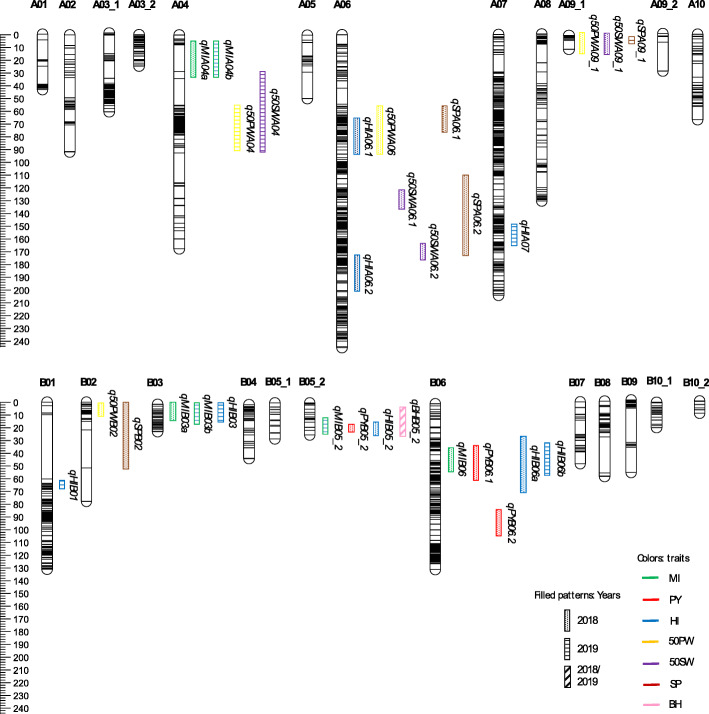
An overview of the genetic map and the QTLs identified for MI and the other traits. MI, maturity index; PY, pod yield; HI, harvest index; 50PW, 50 pod weight; 50SW, 50 seed weight; SP, shelling percentage; BH, branching habit

Genotyping of Hanoch x Harari RILs was performed with version 2 of the Axiom *Arachis*_SNP array consisting of 47 K SNP markers (Thermofisher Scientific). A set of 3283 polymorphic SNP markers between the two parental lines [24] was used in this study. After filtering and removing the missing data and heterozygous calls, 3074 SNPs were retained for the RIL population. Twenty-five RILs with greater than 10% missing data and greater than 20% heterozygous SNP calls were removed from further analysis. Subsequently, a genetic map was constructed with 235 RILs. Also, 773 SNPs that did not obey the chi-square test in the JoinMap tool, 457 SNPs that were identical to other loci, and 11 SNPs that generated final small non-significant linkage groups were excluded.Therefore, the genetic map contained 1833 markers distributed on 24 linkage groups covering a total of 1773.5 cM (Fig. 4; Table 3) (Additional file 2: Table S1).

**Table 3:** Description of genetic linkage groups. Physical distance was determined by blasting markers against the *A. hypogaea* reference genome (peanutbase.org)

Linkage Group	Chromosome assignment	No. of SNPs	Map distance (cM)	Average loci interval (cM)	Physical Length (Mbp)	Average physical interval (Mbp)	Total length *A. hypogaea* genome (Mbp)	Coverage ratio	Recombination rate (cM/Mbp)
A01	Arahy.01	27	43.3	1.6	96.4	3.6	112.42	0.86	0.39
A02	Arahy.02	35	91.7	2.6	96.1	2.7	102.98	0.93	0.89
A03_1	Arahy.03	80	61.9	0.8	39.2	0.5	143.81	0.27	0.43
A03_2		34	24.6	0.7	4.6	0.1	143.81	0.03	0.17
A04	Arahy.04	123	167.4	1.4	124.6	1	128.8	0.97	1.3
A05	Arahy.05	21	49.6	2.4	12.5	0.6	115.93	0.11	0.43
A06	Arahy.06	385	244.6	0.6	115.1	0.3	115.5	1	2.12
A07	Arahy.07	366	203.9	0.6	79.4	0.2	81.12	0.98	2.51
A08	Arahy.08	88	130.7	1.5	50.8	0.6	51.9	0.98	2.52
A09_1	Arahy.09	11	10.8	1	1.7	0.2	120.52	0.01	0.09
A09_2		6	29.3	4.9	99.6	16.6	120.52	0.83	0.24
A10	Arahy.10	38	66.6	1.8	109.9	2.9	117.09	0.94	0.57
B01	Arahy.11	147	130.7	0.9	140.6	1	149.3	0.94	0.88
B02	Arahy.12	29	77.2	2.7	104.2	3.6	120.58	0.86	0.64
B03	Arahy.13	38	21.8	0.6	3.4	0.1	146.73	0.02	0.15
B04	Arahy.14	36	42.7	1.2	124.3	3.5	143.24	0.87	0.3
B05_1	Arahy.15	15	28.2	1.9	112.1	7.5	160.88	0.7	0.18
B05_2		17	25.2	1.5	3.1	0.2	160.88	0.02	0.16
B06	Arahy.16	231	129.9	0.6	131.9	0.6	154.81	0.85	0.84
B07	Arahy.17	23	48.2	2.1	119.7	5.2	134.92	0.89	0.36
B08	Arahy.18	35	59.1	1.7	124.8	3.6	135.15	0.92	0.44
B09	Arahy.19	24	56.8	2.4	129.8	5.4	158.63	0.82	0.36
B10_1	Arahy.20	18	19.9	1.1	127.9	7.1	143.98	0.89	0.14
B10_2		6	9.4	1.6	0.9	0.2	143.98	0.01	0.07
	Mean	76.4	73.9	1.6	81.3	2.8	129.4	0.6	0.7
	Total	1833	1773.5	38.2	1952.6	67.3	3107.5	15.7	16.2

The 24 linkage groups ranged in size from 9.4 cM (B10_2) to 244.6 cM (A06). The average number of loci per linkage group ranged from 76, reaching up to 385 loci in LG A06. The average distance between the neighboring markers was 1.6 cM, ranging from 0.6 cM in LGs A07, A06, B06, and B03 to 4.9 cM in A09_2 (Table 3). Aligning the 1833 markers to the *A. hypogaea* pseudomolecules (peanutbase.org) resulted in a total physical distance of 1952.6 Mbp and an average physical interval of 2.8 Mbp between loci (Additional file 1: Fig. S2; Table 3). The percentage of a pseudomolecule covered by linkage groups varied; eight groups covered more than 80% of a pseudomolecule, seven more than 90%, and one group (A06) was close to 100%. The average recombination rate was 0.7 cM/Mbp. A08 had the maximum recombination rate, while the groups B10_2, A09_1, B03, and B05_2 had the lowest recombination rates.

The linkage map quality was assessed by analyzing the SNPs’ collinearity to their physical positions (Mbp) in the *A. hypogaea* genome (Additional file 1: Fig. S2). As expected, the saturation of the markers in the arms was higher than in the pericentromeric regions. Some rearrangements were exhibited in a few linkage groups, such as apparent inversions in the middle of A07 and at the end of B06 (Additional file 1: Fig. S2).

### QTL identification

QTL mapping of the MI and the other traits resulted in identifying 30 QTLs, with the LOD scores ranging from 3.03 to 81.2, explaining 5.8 to 79.6% of the phenotypic variance (PVE) (Fig. 4; Table 4). Nine linkage groups had at least one QTL, with a maximum of eight QTLs in B06 and 7 QTLs in A06. HI had the maximum number of QTLs, 4 QTLs each in 2018 and 2019. Major QTLs were found for BH and HI, explaining 79.6 and 20.6% PVE, respectively.

**Table 4:** QTL identified for MI and the other traits in the Hanoch X Harari RIL population

Trait	Year	QTL	LG^a^	Position (cM)	Flanking Markers	Physical position range (Mbp)	LOD	PVE (%)^b^	ADD^c^
MI	2018	*qMIA04a*	A04	28.5	AX-176802283_A04 - AX-176815499_A04	117.6–125.59	5.33	9.9	−3.297
MI	2018	*qMIB06*	B06	50.6	AX-147252043_B06 - AX-176807746_B06	11.3–16.6	5.28	9.8	3.335
MI	2018	*qMIB03a*	B03	8.5	AX-176807311_B03 - AX-176806413_B03	2.8–4.6	5	9.3	−3.142
MI	2019	*qMIA04b*	A04	4.3	AX-176819644_A04 - AX-176815499_A04	118.6–125.59	6.45	11.9	−4.917
MI	2019	*qMIB05_2*	B05_2	21.6	AX-147251167_B05 - AX-176821336_B05	156.5–158.9	5.5	10.2	3.689
MI	2019	*qMIB03b*	B03	10.1	AX-176807311_B03 - AX-176801237_B03	2.8–5.7	5.35	9.9	−3.619
PY	2018	*qPYB06.1*	B06	51.2	AX-176806482_B06 - AX-176795044_B06	9.4–24.5	4.92	9.2	140.241
PY	2018	*qPYB06.2*	B06	85.6	AX-176817461_B06 - AX-176797523_B06	46.3–115.9	3.58	6.8	117.604
PY	2018	*qPYB05_2*	B05_2	19.1	AX-147251194_B05 - AX-147251268_B05	157.1–158.3	3.53	6.7	116.586
HI	2018	*qHIB06a*	B06	51.2	AX-176823538_B06 - AX-176793340_B06	7.6–117.1	11.77	20.6	0.075
HI	2018	*qHIB05_2*	B05_2	21.6	AX-147251167_B05 - AX-147223887_B05	156.5–158.4	5.61	10.4	0.051
HI	2018	*qHIA06.2*	A06	180.5	AX-176793198_A06 - AX-177642314_A06	6.7–17.09	5.39	10	−0.051
HI	2018	*qHIA06.1*	A06	75.9	AX-176799874_A06 - AX-176800771_A06	53.9–99.2	5.14	9.6	−0.051
HI	2019	*qHIB03*	B03	5.54	AX-176807311_B03 - AX-176800560_B03	2.8–4.5	5.32	9.9	−0.041
HI	2019	*qHIB06b*	B06	50.0	AX-176819980_B06 - AX-176822996_B06	9.2–22.2	4.71	8.8	0.039
HI	2019	*qHIA07*	A07	150.6	AX-177640658_A07 - AX-176822344_A07	72.6–74.4	3.79	7.2	− 0.035
HI	2019	*qHIB01*	B01	63.7	AX-176797129_B01 - AX-176811427_B01	141.3–145.4	3.03	5.8	0.031
50PW	2018	*q50PWA06*	A06	99.1	AX-176804928_A06 - AX-147225817_A06	10.4–97.2	6.39	11.8	−4.738
50PW	2018	*q50PWA09_1*	A09_1	4.0	AX-176821658_A09 - AX-177644544_A09	118.4–120.1	4.95	9.2	− 4.111
50PW	2018	*q50PWB02*	B02	4.9	AX-147214422_B02 - AX-176813255_B02	103.2–104.7	3.85	7.3	3.684
50PW	2019	*q50PWA04*	A04	81.6	AX-176814690_A04 - AX-147248027_A04	95.3–110.01	4.83	9	3.955
50SW	2018	*q50SWA09_1*	A09_1	4.1	AX-176821658_A09 - AX-177644544_A09	118.4–120.1	5.66	10.5	−1.531
50SW	2018	*q50SWA06.1*	A06	133.9	AX-176805991_A06 - AX-176793602_A06	41.3–73.6	3.35	6.4	−1.229
50SW	2018	*q50SWA06.2*	A06	166.8	AX-176810059_A06 - AX-176816647_A06	16.2–23.2	3.26	6.2	− 1.197
50SW	2019	*q50SWA04*	A04	55.4	AX-176814690_A04 - AX-176819644_A04	95.3–118.6	7.61	13.9	1.316
SP	2018	*qSPB02*	B02	21.6	AX-147239780_B02 - AX-176816518_B02	1.2–3.8	7.73	14.1	−0.793
SP	2018	*qSPA06.1*	A06	62.1	AX-147225784_A06 - AX-176808527_A06	94.0–101.8	4.98	9.3	0.643
SP	2018	*qSPA06.2*	A06	112.5	AX-176816213_A06 - AX-176807067_A06	15.6–75.2	4.87	9.1	0.687
SP	2019	*qSPA09_1*	A09_1	0.3	AX-176821909_A09 - AX-177644544_A09	120.1–120.1	3.08	5.8	0.604
BH	2018/2019	*qBHB05_2*	B05_2	21.6	AX-147251167_B05 - AX-147251374_B05	156.5–159.6	81.2	79.6	−0.456

^a^ *LG* Linkage group; ^b^ *PVE* Phenotypic variance explained; ^c^ *ADD* Additive effect (negative values correspond to the Harari parental line). *MI* Maturity index; *PY* Pod yield; *HI* Harvest index; *50PW* 50 pod weight; *50SW* 50 seed weight; *SP* Shelling percentage; *BH* Branching habit

For MI trait, a total of six QTLs were identified, three QTLs each in 2018 and 2019, respectively (Fig. 4), explaining 9.3 to 11.9% PVE. Two consistent QTL regions were found in both years. One was observed on LG A04 between AX-176819644_A04 - AX-176815499_A04, spanning 6.9 Mbp, with PVE values of 9.9 and 11.9% for 2018 and 2019, respectively. The other consistent QTL region was observed on LG B03 within marker interval of AX-176807311_B03 - AX-176806413_B03, spanning 1.8 Mbp, explaining 9.3 and 9.9% PVE. The other two QTLs were identified on LG B06 (*qMIB06*) and LG B05_2 (*qMIB05_2)*, which were significant only in 2018 and 2019, respectively. Alleles from the early-maturing Harari parent contribute to the high percentage of mature pods measured by MI for four QTLs, *qMIA04a*, *qMIA04b, qMIB03a* and *qMIB03b* (Table 4). The late-maturing Hanoch parent contributed to *qMIB06* and *qMIB05_2*.

For PY, three QTLs were detected in 2018 (Fig. 4; Table 4), two on LG B06 (*qPYB06.1* and *qPYB06.2*), explaining 9.2 and 6.8% PVE, respectively. The other, *qPYB05_2,* was identified on group B05_2, explaining 6.7% PVE. Two of these QTLs, *qPYB06.*1 and *qPYB05_2* overlapped with *qMIB06* and *qMIB05_2* of MI, respectively.

A total of eight QTLs were detected for HI (Fig. 4), explaining 5.8–20.6% PVE. *QHIB05_2* in 2018 shared a common region with *qMIB05_2* in 2019 and *qPYB05_2* in 2018. Another common QTL region shared between *qHIB03* in 2019 and MI trait QTLs, *qMIB03a* in 2018 and *qMIB03b* in 2019 (Table 4). These overlapping QTLs detected between HI and MI traits aligned with the significant correlation between the traits.

For 50PW and 50SW, eight QTLs were identified (Fig. 4). *Q50PWA06* in 2018, *q50SWA06.1* and *q50SWA06.2* in 2018 identified on A06 showed 11.8, 6.4 and 6.2% PVE, respectively. Similarly, *q50PWA04* and *q50SWA04* in 2019 were identified on A04, explaining 9 and 13.9% PVE, respectively. Significant QTLs, *q50PWA09_1* and *q50SWA09_1* were identified in 2018 on LG A09_1 spanning 1.7 Mbp (AX-176821658_A09 - AX-177644544_A09) explaining 9.2 and 10.5% of variation respectively (Table 4). As expected, there was a strong co-localization of QTLs between 50PW and 50SW. An overlapping QTL region was observed in 2019 between *q50SWA04* and MI trait QTLs (*qMIA04a* and *qMIA04b*) spanning 1 Mbp around marker interval AX-176802283_A04 - AX-176819644_A04.

For SP, four QTLs were identified (Fig. 4), three in 2018 and one in 2019. *QSPA06.1* and *qSPA06.2* were identified on A06 in 2018, explaining 9.3 and 9.1% PVE, respectively. Additionally, *qSPB02* in 2018 and *qSPA09_1* in 2019 were observed on B02 and A09_1, explaining 14.1 and 5.8% PVE, respectively (Table 4). An overlapping QTL region was found among *q50PWA09_1* and *q50SWA09_1* in 2018 and *qSPA09_1* in 2019.

For the BH trait, one very significant and consistent QTL, *qBHB05_2* with marker interval AX-147251167_B05 - AX-147251374_B05 on LG B05_2 spanning 3.1 Mbp explaining 79.6% PVE was found. Since almost no differences were found within the same RILs in BH phenotype between 2018 and 2019, the same locus was denoted for both years (*qBHB05_2*). *QBHB05_2* overlapped with the MI QTL, *qMIB05_2* on LG B05_2 in 2019, suggesting a possible BH effect on MI (Fig. 4; Table 4).

## Discussion

Time-to-maturation (TTM) is one of the crucial traits for adaptability and yield in legumes. Late-maturation usually is associated with increased yield and prolonged pod-filling processes [25, 26]. In contrast, early-maturation is associated with better adaptation to terminal stresses and to avoid lodging in some legumes [27, 28]. Genetic factors and their interactions with the environment play crucial roles in the control of TTM. Two main developmental aspects control TTM in legumes, flowering time and plant architecture. Flowering time models in legumes include the vernalization-responsive long-day model and the warm-season short-day model [29]. Inflorescence architecture, the second factor that regulates TTM in legumes, derives from the final identity of the shoot apical meristem [30]. Most legume plants have evolved to a “complex” indeterminate architecture with compound inflorescences [30]. Still, some determinate varieties have been selected in several legumes, such as beans [31], soybeans [32], and peas [33], displaying a shorter flowering time and compact canopy to facilitate mechanized harvesting [34].

Peanut exhibits a unique genetic TTM system for legumes. It is basal to the phaseoloid clade, and therefore should be considered a short-day plant. However, studies clearly show that the time to first flower is minimally affected by photoperiod in peanut [35]. Peanut genotypes typically initiate flowering at about ~ 30 days post sowing regardless of the growing season. Inflorescence architecture systems are more relevant to peanut since the two main cultivated peanut subspecies, *fastigiata* and *hypogaea*, differ in both TTM and flowering patterns. Yet, the lateral shoots of both subspecies have indeterminate growing tips [36], contradicting the “classic” inflorescence architecture system as a possible contributor to differences in TTM between cultivars.

In the current study, we used a unique system to analyze the TTM genetics in peanuts. The segregating RIL population is created from two closely related parental lines that flower at the same time (~ 30 DPP) and have very strong indeterminate growth habits. Additionally, the lines do not differ in the flowering patterns (both have alternate flowering) and branch length, which were previously shown to associate with pod maturity in *fastigiata* peanuts [13]. Other traits that may influence TTM, such as pod number/plant and flowering rate [9, 37, 38], are also irrelevant for this specific population. Therefore, this system is interesting for revealing new genetic components that control TTM in the Virginia-type peanut. Indeed, due to the low genetic variation between Hanoch and Harari, the constructed genetic map seems to have some gaps and low coverage in few chromosomes. However, the two parental lines were part of the 20 genotypes used to construct the Axiom *Arachis*_SNP array, increasing the chance to exploit the best potential of this low polymorphic background.

A significant difference was found between the parental lines in MI in multiple environments. This result agrees with previous observations from commercial fields, showing Harari 12–15 days earlier than Hanoch. The range of MI data in the RIL population extended beyond both parents’ means, suggesting transgressive segregation of MI in this population. Some inconsistency was found between the years in the differences between the parental lines in other traits such as 50SW and PY, partly explained by the relatively small plot size in the experiments. The broad-sense heritability estimates for MI were moderate (~ 0.4) but somewhat higher than reported in other studies [9, 10], demonstrating the relatively strong genetic component and adequate phenotyping in the current system.

The most notable finding in this study was the relatively high phenotypic correlation between MI and harvest index (HI), which was significant in both years. Also, *qMIB03a* and *qMIB03b - qHIB03, qMIB05_2 – qHIB05_2,* and *qMIB06 – qHIB06a* and *qHIB06b* were shared between the traits, indicating a pleiotropic effect. Indeed, the ratio between pod yield biomass and the entire plant biomass at the later stages of development may influence TTM by increasing the plants’ sink capacity and promoting the crop termination. Since the total pod yield (PY) effect was not as strong as HI, we speculate that the canopy biomass had a stronger contribution to TTM than PY. This phenomenon is documented in several legume crops [39–41]. We suggest that sink strength is the most potent effector for early maturation in Virginia-type peanuts, particularly in closely related germplasm, as were used in this study. Interestingly, HI and PY are both quite similar between the parents, although they have distinct MI. In contrast, a relatively large variation in HI and PY was found between the RILs. This suggests that, in addition to MI, other factors determine HI and PY, leading to significant transgressive segregation of these traits in the population.

Another trait that can have some effect on MI in our system is branching habit. Branching habit (BH) is an important descriptive and agronomic character of peanut. In a previous study [42], we showed that the BH in the Hanoch X Harari genetic system is controlled by a single gene that was named *Bunch1*. Using a previous version on the *Arachis* SNP-array, *Bunch1*was located to a ~ 1.1 Mbp segment on the same locus as was found here on B05. So, it is not surprising to find such a high %PVE for BH (Table 4). In the current study, we found that BH has a small but significant effect on MI, particularly in 2019 (Additional file 1: Fig. S1). This is also reflected by the fact that MI and BH shared the same QTL in 2019. The spreading form of BH was associated with higher MI. Interestingly, the allele from the late maturing parental line (Hanoch; spreading) contributed to early maturity at this QTL region. We speculate that pods are formed closer to the ground in the spreading form, and therefore, have the opportunity to mature earlier.

The majority of the QTLs identified have small to moderate effects on MI (Table 4), two of them were consistently detected in both years. Together they explained ~ 20% of the total phenotypic variation for MI and ~ 50% of the genetic variation (taking into account that the heritability estimate was ~ 0.4), indicating that they were not spurious. These two genomic locations are different from QTLs found in other studies [13–15], involving *hypogaea* X *fastigiata* crosses. Thorough literature screening showed that none of the previously described QTL for the other traits in this study (HI, PY, 50SW, 50PW and SP) matched the QTL found here, indicating that they are unique to the Virginia-type background as well.

An indication for the possible influence of these two QTLs in peanut maturation came from a retrospective analysis of the genotypic situation in newly bred Israeli cultivars, ‘Orit’ and ‘Einat’, both originating from similar crosses of Hanoch X Harari, and are presumed to be early-maturing [43]. However, recent multi-location observations of commercial plots have indicated that only Einat is an early maturing variety, while Orit is a more medium-maturing variety. Indeed, inspecting these two varieties’ genotypic information showed that only Einat contains the two consistent QTLs (data not presented). This example illustrates the potential of implementing MAS to provide efficient and unbiased selection of traits with high phenotyping costs such as early- or late-maturity in peanuts.

In conclusion, this study demonstrates the use of SNP-array technology for constructing and applying a genetic map in a biparental population with very low polymorphism. A new genetic map with 1833 SNP markers was constructed for the Virginia peanut background. A total of six QTLs regulating TTM were identified across 2 years of field tests. The novel information and materials generated here can promote the selection of peanut idiotypes using genetic markers associated with the QTLs discovered in this study.

## Materials and methods

### Plant material and growing conditions

A recombinant inbred line (RIL) population was developed from a cross between cv. Hanoch and cv. Harari [44], two closely related Israeli Virginia-type cultivars differing in TTM (Fig. 1a). RILs were obtained by a single seed descend procedure, up to F7, and then were multiplied as bulks for additional two generations (F7:9). ‘Hanoch’ has been the leading Israeli in-shell peanut cultivar for over two decades. It is a late-maturing cultivar with long, smooth, and hard pods. These qualities make it well received in the EU “in-shell” market. ‘Harari’ is an early-maturing cultivar grown in Israel’s northern part. Its growing season is limited by late sowing time (due to the double-cropping system) and early harvest (due to autumn rainfalls). Harari has reticulated and soft pod walls and is targeted for the local shelled industry. Harari has a bunch-type growth habit while Hanoch is spreading. Common traits between Hanoch and Harray include pod size, flowering time, flowering pattern, lateral branch length, pod number/plant and flowering rate. Both parental lines were part of the *Arachis*_SNP-array development panel [45].

A total of 260 RILs were planted in two successive years, respectively. The first year was planted in April 2018 in the Hula Valley, Northern Israel (33°11′17.7″N 35°34′25.6″E), characterized by heavy black soil. The second was planted in April 2019 in Urim, Western Negev, Israel (31°20′27.4″N 34°29′46.1″E), characterized by fine sandy-loam. Besides the soil type, these two regions are significantly different in environmental conditions. Hulla Vally is a typical semi-arid climate with rainy winters and high summer humidity, while Nirim is located in the Negev Desert with low humidity. A similar experimental design of randomized complete blocks with three replications was implemented in both seasons. Each ‘plot’ (line X block) consisted of two rows on a bed, 4 m in length, rows spaced 90 cm apart, and seeding rates of 10 seeds/m^2^ (total of 20 plants/plot). Parental lines were grown as control plots with nine replications. Fields were maintained under full-irrigation conditions, and all recommended agronomic practices were carried out as previously described [44]. All plant material, including the parental and the RILs, was originated from Hovav laboratory and there are part of the ARO breeding program.

#### Phenotyping the maturity trait and post-harvest traits

TTM was evaluated at ~ 140–145 days post-planting (DPP). The exact sampling date was determined by testing the parental lines every few days, starting at 125 DPP, up to the point where Harari was ~ 60% mature on average. This timing was chosen in order to capture the widest variation in maturation among the RILs. The hull-scrape method [23] was used to measure the maturity level by randomly sampling 2–3 plants per plot and removing the exocarp from all pods using a PICO water pressure machine (Idromatic®, Italy), 14 MPa with 9 l/minute flow rate. Pressure-washed pods were separated into five categories based on mesocarp color: white, yellow, orange, brown, or black. Pod number in each category was documented, and the maturity index (MI) was calculated as the percentage of pods in the brown and black categories. In total, 729 and 780 MI measurements were taken for 2018 and 2019, respectively.

Other traits potentially associated with TTM were also recorded. Branching habit (BH) was documented at ~ 50 DAP as a spreading or a bunch. Negligible variations were found between 2018 and 2019 for BH data. Therefore, one value was used for BH in both years. After digging, plots were dried for 7–10 days, and the entire biomass of each plot (excluding the tap-roots) was weighed. Each plot was then threshed by an experimental thresher (Kincade, USA), and total pod yield/plot (PY) was measured. Harvest index (HI) was calculated as the ratio of pod yield/plot over biomass weight/plot. 50-pod weight (50PW), 50-seed weight (50SW), and shelling percentage (SP) were recorded by randomly sampling 100 pods from each plot.

### Statistical analysis of phenotypic data

Statistical difference between the parents was determined by Student’s t-test. As for the RILs, Anderson-Darling test was performed to determine the normality of distribution. The ANOVA analysis model included the RIL, Year, Year X RIL and Block [Year] effects. All effects were defined as random to calculate the heritability rates. Broad sense heritability (*H*^2^) was estimated with the equation *H*^*2*^ = σ_g_^2^/(σ_g_^2^ + σ_ge_^2^ + σ_e_^2^), by the ANOVA analysis with QTL IciMapping v4.2 (http://www.isbreeding.net/software/?type=detail&id=29) [46]. The σ_g_^2^, σ_e_^2^ and σ_ge_^2^ denoted the variances of genotypes (G), environment (E) and interaction of genotypes and environments (G x E). Correlation coefficients were calculated among all the traits across years. One way ANOVA was performed to check the effect of the BH phenotype on MI. Distribution and correlation statistics, histograms, and boxplots were performed with JMP® Pro 15 (SAS Institute Inc., Cary, NC, 1989–2019).

### Genotyping and genetic map construction

Genomic DNA was extracted using DNeasy® Plant Mini Kit (Qiagen; Hilden, Germany) from young leaflets from each RIL and the two parents. DNA quantification was performed with Qubit (Invitrogen; CA, USA). The samples were diluted to 40 ng/μL according to protocol guidelines and genotyped by using the Affymetrix Axiom_Arachis2 SNP array comprising 47,837 SNPs, divided into their AA and BB subgenomic origin [20, 47]. Genotyping data were analyzed by the Axiom analysis suite Software 3.1 [24]. The polymorphic homozygous SNPs (AA and BB) and polymorphic heterozygous SNPs (AA or BB and AB) were retained with 65–35% call-rate frequencies among the RILs. Out of 260 RILs, 25 RILs with greater than 10% missing data and greater than 20% heterozygous SNP calls were removed from further analysis. Subsequently, the genetic map was constructed with 235 RILs. The genetic linkage map was constructed using Joinmap v4.1 [48] maximum likelihood (ML) algorithm with a minimum LOD of 3.0 and the Haldane mapping function. The graphical representation of the linkage maps was generated through Mapchart v2.3 [49]. Confirmation of the loci positions was done as previously described [47] with few modifications (BLASTN (e value < 1 × 10^− 18^) and mismatch of less than 2). Linkage groups (LG) generated were assigned to the pseudo-molecules of the tetraploid *A. hypogaea* cv. Tifrunner [17] (https://peanutbase.org). Due to the high sequence similarity between the two subgenomes of cultivated peanut [16], the position assignment of the 70-bp-long SNP markers from the array can be ambiguous. Linkage groups were assigned to the respective pseudomolecules (chromosomes) of the sequenced *A. hypogaea* genome cv. Tifrunner (Table 3). If a LG had more than 51% of the SNPs representing a particular chromosome, then this LG was assigned to that chromosome. To assess the quality of the genetic map, a collinearity analysis was performed using the genetic distances (cM) versus the physical positions (Mbp).

### QTL analysis

QTL mapping was performed on 235 RILs using MapQTL v6 [50] on the mean phenotypic data collected each year (Additional file 2: Table S2). A LOD score of 3 with 1000 permutations was used to confirm the presence of a putative QTL at a 95% significance level. QTLs were manually assigned to the genetic maps. The naming of the QTL follows the terminology of “*q*” as QTL, followed by an abbreviation of the trait. The last digit represents the LG, and repetition of the QTL in alphabetical order if in both years, or numerical order if there are more than one, on the same LG. SNP markers flanking the QTL were used to obtain the physical position from the *A. hypogaea* genome.

## Supplementary Information

**Additional file 1: Fig. S1.** Box plot analysis to study the effect of branching habit (X-axis) on maturity index (Y-axis) across 2018 and 2019. *P* values were generated through Student’s t-test. The color of the boxes indicates as follows, blue, bunch; red, spreading. **Fig. S2.** Correlation between the genetic distance (cM) (x-axis) of markers on each linkage group (LG) and the physical genome position (Mbp) (y-axis) based on the Tifrunner reference genome. Black dots represent markers mapped to the respective chromosome, red dots indicate markers mapped to the homeologous chromosome and black circles represent markers mapped to other chromosomes.

**Additional file 2: Table S1.** Population genotype data presented by the order of linkage map. Physical positions of markers were based on the tetraploid peanut genome sequence [17]. **Table S2.** Mean phenotypic values of measured traits over two years of field tests.

## Data Availability

The data sets supporting the results of this study are included in the manuscript and additional supporting files.

## Abbreviations

50PW: 50-pod weight
50SW: 50-seed weight
ADD: Additive effect
HI: Harvest index
LG: Linkage group
LOD: Logarithm of the odds
MI: Maturity index
PY: Pod yield
PVE: Phenotypic variation explained
QTL: Quantitative trait locus
RIL: Recombinant inbred line
SP: Shelling percentage
SNP: Single nucleotide polymorphism
TTM: Time to maturation
