# Supplementary material for: Identification of consistent QTL for time to maturation in Virginia-type Peanut (Arachis hypogaea L.)
Source: BMC Plant Biol. 2021 Apr 19;21:186. doi: 10.1186/s12870-021-02951-5 (PMC8054412; doi:10.1186/s12870-021-02951-5)
Supplement: Supplementary file 1 — Additional file 1: Fig. S1. Box plot analysis to study the effect of branching habit (X-axis) on maturity index (Y-axis) across 2018 and 2019. P values were generated through Student’s t-test. The color of the boxes indicates as follows, blue, bunch; red, spreading. Fig. S2. Correlation between the genetic distance (cM) (x-axis) of markers on each linkage group (LG) and the physical genome position (Mbp) (y-axis) based on the Tifrunner reference genome. Black dots represent markers mapped to the respective chromosome, red dots indicate markers mapped to the homeologous chromosome and black circles represent markers mapped to other chromosomes. [file 12870_2021_2951_MOESM1_ESM.docx]

**Identification of Stable QTL for Time to Maturation in Virginia-Type Peanut (*Arachis hypogaea* L.)**

Srinivas Kunta^1,2^, Sara Agmon^1^, Ilan Hedvat^1^, Yael Levy^1^, Ye Chu^3^, Peggy Ozias-Akins^3^ & Ran Hovav^1*^

**Affiliations**

^1^ Department of Field Crops, Institute of Plant Sciences, Agriculture research organization-The Volcani Center, HaMakkabbim Road, P. O. Box 15159, 7505101 Rishon LeZiyyon, Israel. ^2^ Faculty of Agricultural, Food and the Environmental Quality Sciences, the Hebrew Univ. of Jerusalem, POB 12, Rehovot 76100, Israel. ^3^ Department of Horticulture and Institute of Plant Breeding, Genetics and Genomics, University of Georgia, Tifton, GA 31793 USA

**Fig. S1** Box plots analysis to study the effect of branching habit (x-axis) on maturity index (y-axis) across 2018 and 2019. *P* values were generated through Student's t-test. The color of the boxes indicates as follows, blue, bunch; red, spreading.

Physical Position (Mbp)

Genetic distance (cM)

**Fig. S2** The correlation between the genetic distance (cM) (x-axis) of markers on each linkage group (LG) and the physical genome position (Mbp) (y-axis) based on the Tifrunner reference genome. Black dots represent the respective chromosome markers with the LG, red dots to the homologous chromosome and black circles represent the markers from other chromosomes.
